# Spontaneous tumours in dogs: A clinical and pathomorphological study in Kyrgyzstan

**DOI:** 10.17221/16/2024-VETMED

**Published:** 2024-06-25

**Authors:** Svetlana Ishenbaeva, Rysbek Nurgaziev, Urmatbek Tynaliev, Uranbek Shergaziev, Almazbek Irgashev

**Affiliations:** ^1^Department of Veterinary and Sanitary Expertise, Histology and Pathology, Kyrgyz National Agrarian University named after K.I. Skryabin, Bishkek, Kyrgyz Republic; ^2^Silk Road Research Center, Ala-Too International University, Bishkek, Kyrgyz Republic

**Keywords:** dogs, clinical study, histopathology, localisation, tumours

## Abstract

The aim of this study is to investigate the correlation between the incidence of spontaneous tumours of various origins and the localisation in dogs with sex, breed, and age factors. A total of 360 tumours with various localisation were studied pathomorphologically. Histopathologic data sets from 360 dog tissue samples were processed and statistically examined. A chi-square test of independence was conducted to examine the relationships among the various levels of the specified variables. Logistic regression models were employed for dichotomous outcomes to ascertain the influence of certain explanatory variables on the tumour types. Characteristic pathomorphological changes observed during examination of dogs with oncologic diseases were determined. The most common neoplasms were mammary tumours, accounting for 43% of the cases. The mammary gland tumours were most common in mongrel dogs (25%), with German Shepherds (17.3%), Poodles, Dachshunds, Central Asian Shepherds (6.7% each), and Rottweilers (5.7%) following. The highest frequency of these tumours appeared at 8 years of age, predominantly originating from the ductal epithelium, which represented 46.4% of all the malignant cases.

The incidence of cancer is increasing globally in both humans and animals. This trend underscores a significant challenge in modern medical research, which is often hampered by the complex biology of cancer (Ciaputa et al. 2022). Notably, the histological and genetic similarities between canine and human tumours position dogs as valuable models for studying cancer in humans (Schiffman and Breen 2015). Both species are in direct contact with similar environmental conditions and are exposed to comparable carcinogens. Such exposures, alongside genetic, epigenetic, and metabolic changes, contribute to the development of cancer in both dogs and humans (Oh and Cho 2023).

In dogs, tumours are most commonly diagnosed between 8 and 10 years of age. This age range approximately corresponds to the human age of 50–60 years, suggesting that, as in humans, canine cancer may be influenced by age and environmental factors (Cekanova and Rathore 2014). Additionally, the shorter lifespan of dogs, coupled with the generally rapid progression of cancer in this species, facilitates the timely assessment of clinical outcomes. This characteristic also aids in the early detection of adverse environmental and carcinogenic factors that may be relevant to humans (Bronden et al. 2007). Moreover, certain cancers that are rare in humans, such as osteosarcoma and haemangiosarcoma, occur with greater frequency in dogs. This situation allows scientists to study these cancers more in dogs, which could help us learn ways to treat them in both dogs and humans.

In this study, we looked at how often different cancers occur in dogs, and whether things like the dog’s sex, breed, and age affect this. We identified patterns that may explain the reasons behind the higher prevalence of cancer in certain dog populations, as well as the specific anatomical locations most affected. Understanding these patterns can help in making better treatments for cancer in dogs and might even give us ideas for treating cancer in humans.

The materials and results of this study enhance our understanding of tumours in dogs. Additionally, they provide a basis for developing diagnostic measures for canine cancer.

## MATERIAL AND METHODS

### Sample selection

The study utilised neoplasm samples obtained from several veterinary clinics in Bishkek. For the histological analysis, the samples were collected through incisional and excisional biopsies during the surgical excision of neoplasms in dogs of various ages and breeds.

All the surgically removed organs and tissues, including the obtained material, were fixed in a 10% aqueous solution of neutral formalin. Paraffin embedding was employed to prepare the histological sections. Sections of 5–6 μm thickness were produced using a sledge microtome, specifically the modified pfmSlide 4004M model. The sections were then stained using the haematoxylin and eosin method. Histological preparations were examined using a Leica DM 750 microscope at various magnifications (× 40, × 100, and × 400). These preparations were microphotographed with ToupCam UCMOS01300KPA digital cameras. The diagnosis of neoplasms was performed by pathomorphologists from the scientific laboratory of the Department of Veterinary and Sanitary Examination, Histology, and Pathology at the Kyrgyz National Agrarian University named after K.I. Skryabin. For the histological verification of the tumours, the international classification of animal tumours was employed (Valli 2007).

The diagnosis of tumours in dogs was based on an analysis of data from outpatient registers, including morphological findings and the results from laboratory and diagnostic studies. These analyses were conducted according to a specified algorithm and the rules of specialised veterinary admission. In some cases, additional diagnostic methods, such as radiography and diagnostic laparotomy, were employed.

When collecting anamnestic data, we considered various factors regarding the living conditions of the dogs, including their maintenance, feeding habits, past diseases, surgical interventions, and pedigree. Additionally, we gathered detailed histories of the disease, encompassing the onset, course, and development of neoplasms, as well as the tumour growth rate, based on the observations reported by the owners. Upon detecting a tumour, we assessed several characteristics: its localisation, size, growth pattern, consistency, and mobility relative to the surrounding tissues. We also evaluated the body’s response to the tumour, focusing on signs of inflammation and the condition of the lymph nodes.

### Statistical analysis

A chi-square test of independence was conducted to examine the relationships among the various levels of the specified variables as shown in subsequent sections. Logistic regression models were employed for the dichotomous outcomes to ascertain the influence of certain explanatory variables on the tumour types. All the analyses were carried out using the Stata software (StataCorp LLC v16, 2019). The threshold for statistical significance was set at *P* < 0.1.

## RESULTS

The classification of tumours based on the age, sex, and malignancy provided insights from an analysis of 360 canine tumour samples. The age of the dogs examined ranged from 1 month (0.1 years) to 18 years, with an average age of 7.8 years and a standard deviation of 3.7 years. Notably, in the youngest age group (0–3 years), the incidence of malignant tumours, primarily skin tumours and lymphoproliferative diseases, was significantly higher than that of benign tumours.

Among the sampled population, 247 dogs (68.6%) were female, while 113 dogs (31.4%) were male. Regarding tumour malignancy, 166 samples (46.1%) were classified as malignant, and 194 samples (53.9%) were benign.

A total of 360 tumours in dogs, representing 9.2% of the total number of examined dogs, were pathomorphologically studied. Among these, 46.1% (*n* = 166) were malignant, and 53.9% (*n* = 194) were benign. In terms of the tumour localisation, the most commonly affected areas were the mammary gland tumours and skin, accounting for 43% (*n* = 155) and 31.6% (*n* = 114) of cases, respectively. In contrast, tumours of internal organs were found in only 2.5% of cases.

Among the dogs affected by spontaneous tumours in our study, 68.6% were females and 31.4% were males, accounting for 247 females and 113 males in a total group of 360 dogs with neoplasms. Specifically, 43% of all the dogs with tumours were affected by mammary gland tumours. In contrast, skin tumours were found in 70 males (24.9%) and 44 females (15.6%).

Since the veterinary clinics received dogs of various breeds, we analysed the influence of the breed on the tumour frequency. Among the dogs with spontaneous tumours, 78% were purebred, while 22% were mongrels. The most common breeds among purebred dogs were: German Shepherds (15.8%), Central Asian Shepherds (7.7%), Rottweilers and Dachshunds (5.8% each), Toy Terriers (4.1%), Poodles and Shar Peis (3.8% each), and Boxers and Pekingese (2.7% each).

**Figure 1:**
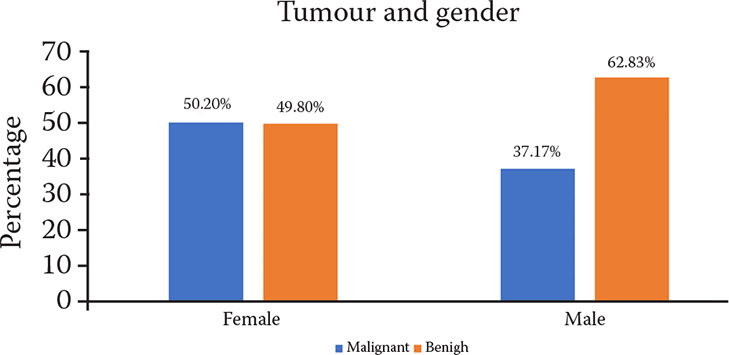
Tumour type and sex correlation

The correlation between the tumour type and sex was found to be significant, χ²(1, *N* = 360) = 5.30, *P* = 0.021; Cramér’s *V* = 0.12. As illustrated in Figure 1, male dogs have a higher prevalence of benign tumours (almost 63%) compared to female dogs, which exhibit an almost equal distribution of both benign and malignant tumours. Regarding Cramér’s *V* = 0.12, the effect size measurement for the chi-square test indicates a weak, but statistically significant association that was found between the tumour type and sex in the studied canine population.

### Tumour characteristics according to the localisation

**Figure 2:**
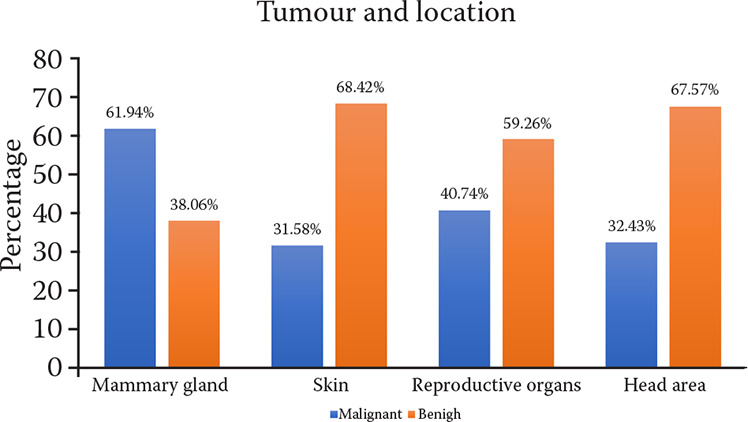
Tumour type and location correlation

The relationship between the tumour type and localisation was found to be significant, χ²(3, *N* = 360) = 28.72, *P* < 0.001; Cramér’s *V* = 0.28. Tumour glands were more likely to appear in certain areas of the dogs’ bodies. For example, the distribution of tumors in the overall canine population mammary glands tumours accounted for 43% of the cases, the skin tumours accounted for 32%, reproductive organs tumours accounted for 15%, and tumours in the head areas accounted for 10% (Figure 2). Additionally, the effect size measurement for the chi-square test, represented by Cramér’s *V* of 0.28, indicates a moderate correlation, further substantiating the strength of this relationship.

As indicated in Table 1, four explanatory categorical variables were analysed in relation to the type of tumour, categorised dichotomously as malignant or benign. The analysis suggests that the presence of a second or recurrent tumour may have a protective effect, potentially reducing the risk of the tumour formation by 42% (OR, 0.58; 95% CI, 0.32–1.04; *P* = 0.06). However, the breed size, especially average (OR, 2.37; 95% CI, 1.14–4.91; *P* = 0.021) and large (OR, 2.08; 95% CI, 1.00–4.33; *P* = 0.049) sizes, are associated with an increased risk of tumours. Regarding the location of the tumours, the skin (OR, 3.21; 95% CI, 1.86–5.55; *P* < 0.001) exhibited higher susceptibility to tumours compared to other locations, including the mammary gland. Additionally, reproductive organs (OR, 2.37; 95% CI, 1.21–4.65; *P* = 0.012) and head regions (OR, 2.38; 95% CI, 1.07–5.29; *P* = 0.032) also showed a higher likelihood of tumour development. The age was found to have no significant effect on the development of tumours in domestic dogs.

**Table 1:** Multivariable logistic regression of the variables associated with the tumour in domestic dogs (pets)

Variables	OR (95% CI)	β	Robust SE	*Z*-value	Pr(>|*z*|)
First	–	–	–	–	–
Repeat	0.58 (0.32–1.04)	–0.54	0.29	–1.83	0.06*
Age	0.95 (0.88–1.01)	–0.05	0.03	–1.58	0.11
Small	–	–	–	–	–
Average	2.37 (1.14–4.91)	0.86	0.37	2.31	0.021**
Big	2.08 (1.00–4.33)	0.73	0.37	1.97	0.049**
Mammary gland	–	–	–	–	–
Skin	3.21 (1.86–5.55)	1.17	0.28	4.19	0.00***
Reproductive organs	2.37 (1.21–4.65)	0.86	0.34	2.52	0.012**
Head area	2.38 (1.07–5.29)	0.87	0.40	2.14	0.032**
Intercept	–	–0.58	0.45	–1.27	0.205

*P < 0.1, **P < 0.05, ***P < 0.01

β = standardised regression coefficient; CI = confidence interval; OR = odds ratio; SE = standard error

In Table 2, which evaluates the recurrence of tumours in domestic dogs and the influence of specific categorical explanatory variables, the following findings were observed. Dogs diagnosed with haemoblastoses had the highest risk of tumour recurrence (OR, 5.15; 95% CI, 1.53–17.28; *P* = 0.008). Regarding the localisation of the tumours, those in the reproductive organs (OR, 0.11; 95% CI, 0.03–0.38; *P* < 0.001) and head areas (OR, 0.06; 95% CI, 0.01–0.46; *P* = 0.007) had a higher likelihood of recurrence compared to mammary gland tumours. In contrast, tumours located in the skin (OR, 0.43; 95% CI, 0.19–1.03; *P* = 0.058) were less likely to recur, with a decreased likelihood of approximately 57%. Additionally, the presence of multiple tumours (OR, 1.97; 95% CI, 1.09–3.54; *P* = 0.023) increased the likelihood of recurrence. However, factors such as the age, sex, and tumour type did not significantly affect the recurrence of tumours in domestic dogs.

**Table 2:** Multivariable logistic regression of the variables associated with the first/repeat occurrence of tumours in domestic dogs (pets)

Variables	OR (95% CI)	β	Robust SE	*Z***-**value	Pr(>|*z*|)
Epithelial tumours	–	–	–	–	–
Mesenchymal tumours	0.89 (0.45–1.75)	–0.11	0.34	–0.32	0.75
Haemoblastoses	5.15 (1.53–17.28)	1.64	0.62	2.66	0.008***
Single	–	–	–	–	–
Multiple	–	–	–	–	–
Mammary gland	–	–	–	–	–
Skin	0.43 (0.19–1.03)	–0.82	0.43	–1.90	0.058*
Reproductive organs	0.11 (0.03–0.38)	–2.17	0.61	–3.56	0.00***
Head areas	0.06 (.008–0.46)	–2.82	1.05	–2.69	0.007***
Malignant	–	–	–	–	–
Benign	0.65 (0.37–1.14)	–0.43	0.28	–1.50	0.134
Female	–	–	–	–	–
Male	1.88 (0.78–4.52)	0.63	0.45	1.42	0.157
Intercept	–	–1.00	0.25	–3.98	0.00

*P < 0.1, **P < 0.05, ***P < 0.01

β = standardised regression coefficient; CI = confidence interval; OR = odds ratio; SE = standard error

### Mammary gland tumours in dogs

Mammary gland tumours represent a prevalent oncological condition, accounting for 43% (*n*** = **155) of all the tumours in the dogs we examined. Upon histological examination, 27.8% of these cases were diagnosed as benign, while 67.3% were malignant mammary gland tumours. The highest incidence of these tumours was observed in dogs aged 6 to 9 years.

These animals were predominantly unsterilised or sterilised at an advanced age. A notable history of pseudopregnancy was frequently observed in dogs. Among the breeds, mammary gland tumours were most commonly diagnosed in mongrel dogs (25%) and German Shepherd dogs (17.3%). Mammary gland tumours are commonly diagnosed in female dogs (Salas et al. 2015; Silva et al. 2019). Nevertheless, this study also examined four male dogs presenting with mammary gland neoplasms. Among them, a 2.5-year-old male Rottweiler was diagnosed with a soft fibroma of the mammary gland. Due to the insufficient sample size, these male cases were not included in the further analysis of the study.

Clinically, we categorised mammary gland cancer in dogs into two forms: nodular (47.7%) and diffuse (37.3%). In the nodular form, the cancer presented as tumour nodules of varying shapes and sizes with clear borders and a dense consistency. In contrast, the diffuse form typically involved dense masses spreading from either the third or the first gland to the fifth gland on one or both sides. Nodular cancers often exhibited slow, long-lasting growth, while diffuse cancers were characterised by more rapid growth.

The histological structure of the mammary gland tumours we examined was highly diverse. Most commonly, cancer developed from the ductal epithelium, accounting for 46.4% of all the malignant mammary gland tumours.

Mammary gland tumours were histologically diagnosed according to the 2011 histological classification standards by Goldschmidt et al. (2011), whose approaches were further developed by Pena et al. (2013). Among benign tumours, fibroadenomas/fibromas and benign mixed tumours predominated: 13.5% (21/155) and 7% (11/155), respectively. Among malignancies, solid carcinoma was observed in the highest proportion at 13.5% (21/155), followed by simple carcinoma and complex carcinoma at 10% (16/155) and 8% (13/155), respectively. Among tumour-like neoplasms of the mammary glands in dogs, in the vast majority of cases, we observed nodular fibrocystic mastopathy with varying degrees of proliferation of the epithelium and connective tissue, hyperplasia and dysplasia of cellular elements (Table 3).

**Table 3:** Histopathological classification of the mammary tumours in the dogs in Bishkek (*n*** = **155)

1.	Malignant epithelial neoplasms	
	1.1. Carcinoma – *In situ*	2
	1.2. Carcinoma – Simple	16
	Tubular	9
	Tubulopapillary	8
	Cystic-papillary	2
	1.3. Carcinoma – Solid	21
	1.4. Carcinoma – Anaplastic	1
	1.5. Carcinoma – Complex type	13
2.	Malignant epithelial neoplasms – Special types	
	2.1. Squamous cell carcinoma	4
	2.2. Adenosquamous carcinoma	3
	2.3. Mucinous carcinoma	2
3.	Malignant mesenchymal neoplasms – Sarcomas	
	3.1. Osteosarcoma	3
	3.2. Chondrosarcoma	3
	3.3. Fibrosarcoma	4
	3.4. Hemangiosarcoma	1
	3.5. Other sarcomas	4
Subtotal:		96
4.	Benign neoplasms	
	4.1. Adenoma – Simple	7
	4.2. Intraductal papillary adenoma	4
	4.3. Fibroadenoma/fibroma	21
	4.4. Benign mixed tumour	11
Subtotal:		43
5.	Hyperplasia/dysplasia	
	5.1. Lobular hyperplasia (adenosis)	6
	5.2. With fibrosis – Interlobular fibrous connective tissue	4
	5.3. Papillomatosis	3
	5.4. Fibroadenomatous change	3
Subtotal:		16
Total:		155

**Figure 3:**
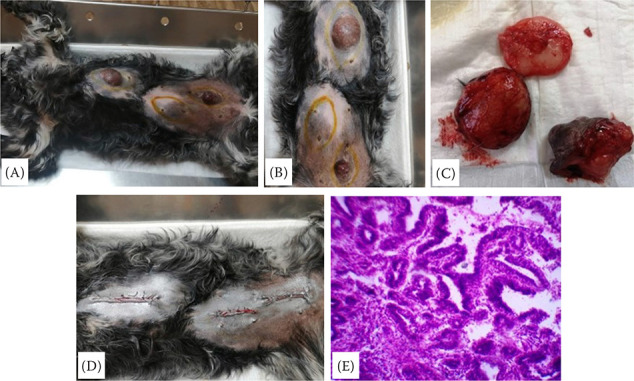
Dog of a Russian Spaniel breed. 10 years old (A–D) Macroscopic lesion of the mammary gland with fibroadenoma. (E) Histologic picture of a highly differentiated adenocarcinoma. Haematoxylin&eosin staining. Eq.** × **100

To illustrate our findings, we present photos of the macroscopic and histological changes in a moderately differentiated adenocarcinoma as an example (Figure 3).

An autopsy was performed on a dog cadaver presenting with a neoplasm, followed by a subsequent histologic examination. The examination of the cadaver revealed a neoplasm located in the area of the mammary glands. The tumour was round, measuring 6** × **9 cm in diameter. The surrounding skin was stretched, and the nodule’s consistency was dense, with its base tightly fused to the tumour tissue. Upon making a transverse section, we observed greyish-white areas alongside regions of dense tissue and cystic cavities of varying sizes, filled with a light brown fluid.

**Figure 4:**
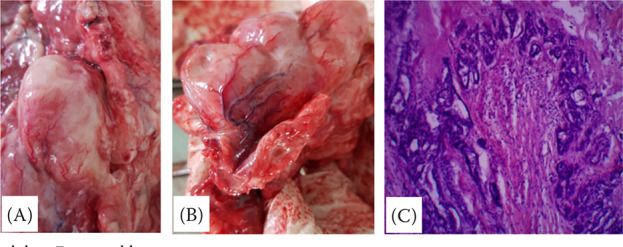
Mongrel dog, 7 years old (A,B) Macroscopic skin lesion in the breast area of a highly differentiated adenocarcinoma. (C) Histologic structure of a highly differentiated adenocarcinoma of the breast. Haematoxylin&eosin staining. Eq.** × **40

Dense white growths with a soft consistency were detected on the inner part of the tumour, which occupied almost the entire lower abdominal wall. Vessels had formed inside the cystic cavities (Figure 4A,B). The histologic examination revealed small gland-like structures and nested clusters and strands of cells, showing pronounced signs of atypism (Figure 4C).

### Skin tumours in dogs

In our study on the prevalence of tumour diseases in dogs, skin tumours were diagnosed in 114 dogs, accounting for 31.6% of all the tumours. Among these, benign skin tumours comprised 68.42% and malignant tumours 31.58% as indicated in the Figure 2. The age range of dogs with skin tumours was from 1 to 15 years, with the majority (33.3%) being aged between 6 to 9 years. These neoplasms were observed in various breeds, with purebred dogs constituting 88.6% and mongrel dogs 11.4%. Among the purebreds, German Shepherd dogs (20%), Central Asian Shepherd dogs (8.7%), and Shar Peis (7.8%) were the most commonly affected.

**Figure 5:**
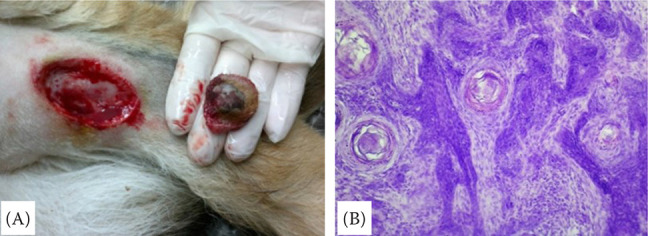
Dog of the German Shepherd breed. 7 years old (A) Macroscopic skin lesion near the hamstring lymph node for a squamous cell keratinising cancer. (B) Histologic change in a squamous cell keratinising skin cancer. Haematoxylin&eosin staining. Eq.*** × ***100 1 – infiltrative growth of epithelial masses; 2 – cancer “pearls”

The skin neoplasms typically appeared as dense nodules, pale pink or grey in section. Tumours were diagnosed using clinical and pathomorphological methods. Regarding their localisation, most skin neoplasms in dogs were found on the trunk (61.6%), followed by the limbs (19.3%), the eye area (9.6%), and the head (7%). As an illustrative example, we present photographs showing macroscopic and histological changes in a case of a squamous cell keratinising skin cancer in a dog. Within the scope of our investigation focused on canine health, we identified 114 cases of skin tumours. Among these, 65% were classified as epithelial tumours. The breakdown of epithelial neoplasms included tumour-like formations (30%), squamous cell carcinoma (10.5%), basal cell carcinoma (7.9%), papilloma (7.8%), and sebaceous gland adenoma (7%). Rare occurrences involved sebaceous gland cancer and epithelioma. For non-epithelial tumours, our findings were fibroma (14%), haemangioma (7%), melanoma (5.2%), neurilemmoma, and trichofolliculoma (1.75%), with liposarcoma, leiomyoma, and lipoma presenting in isolated cases. Cutaneous lymphomas were diagnosed in five cases (4.3% of all the skin tumours), notably three dogs were under 2 years (Figure 5).

## DISCUSSION AND CONCLUSION

The aim of this study was to characterise the clinical and pathomorphological changes in the organs and tissues of dogs with neoplastic diseases and to establish clinical and morphological parallels based on these findings. Additionally, this study sought to examine the relationship between the incidence of spontaneous tumours of various origins and the localisation in dogs and factors such as the sex, breed, and age.

In the current study, among the 360 pathomorphologically confirmed cases of neoplasia, 46.1% were malignant, and 53.9% were benign. These findings align with other studies where benign tumours are more prevalent than malignant ones (Pinello et al. 2022b; Smiech et al. 2023). According to the majority of researchers, skin tumours are the most common in dogs, accounting for 23% to 46% of all tumours (Hauck and Oblak 2020). However, in our study, mammary tumours were predominant, comprising 43% of the cases, followed by skin tumours at 31.6%. This discrepancy might be attributed to the veterinary clinic’s tendency to send fewer samples of benign skin tumours for morphological study at the university.

We diagnosed spontaneous tumours in dogs across different age groups, with the mean age of the affected animals being approximately 7.8 years. These results concur with findings reported by other authors (Pinello et al. 2022a). Among the dogs affected by tumours in our study, 68.6% were females, and 31.4% were males. The higher proportion of females with spontaneous tumours can be attributed to the high incidence of mammary gland neoplasms, which accounted for 43% of all the tumours in our canine subjects. This prevalence of spontaneous tumours in female dogs, ranging from 50% to 70%, is also reflected in the works of other researchers (Martins et al. 2022).

Also, large breed dogs were found to be more predisposed to cancer pathology, with German Shepherds accounting for 15.8% and Central Asian Shep-herds for 7.7%. Breeds such as Bulldogs, Dalmatians, Taigans, St. Bernards, and Riesenschnauzers demonstrated a lower risk of tumour development. This contrasts with the findings of Dobson (2013), where German Shepherd dogs were reported to have a low risk of tumour development. Other authors have identified breeds like Boxers, Golden Retrievers, St. Bernards, and Riesenschnauzers as more susceptible to tumours (Kok et al. 2019). The variations in results between different studies could be attributed to the differences in the breed population sizes in the respective study locations. Consequently, the data regarding the impact of breed on tumour development are diverse, underscoring the need for further research to elucidate this relationship.

Our findings regarding skin tumours are consistent with other reports, with skin neoplasms accounting for 31.6% of all the tumours in the dogs we examined. Among these, benign skin tumours comprised 68.42% and malignant tumours 31.58%. This is in line with findings by Smiech (2023), Martins et al. (2022), and Gruntzig et al. (2015), where benign tumours predominated in skin neoplasms, up to 65%, while some studies, like those referenced by (Baioni et al. 2017), reported a higher prevalence of malignant tumours (56.6%).

The skin tumours were diagnosed in dogs aged 1 to 15 years, with the majority (33.3%) being between 6 to 9 years old. These results align with other studies that report the occurrence of skin neoplasms in dogs primarily at the age of 7–10 years (Pakhrin et al. 2007). Kok et al. (2019) noted that dogs aged 11 years and older are at a significantly increased risk of developing skin tumours. Among the 114 skin neoplasms we identified, 65% were epithelial tumours, compared to the 56% prevalence reported in Pakhrin’s 2007 study. Additionally, squamous cell carcinoma was the most common malignant skin tumour we diagnosed in dogs (10.5%), whereas Kok et al. (2019) found soft tissue sarcomas to be more prevalent (18.40%).

In previous studies, mammary tumours typically occurred in middle-aged and older dogs, with an increased risk noted between 8 and 11 years of age (Kim et al. 2017). Furthermore, benign tumours were more frequently observed in dogs aged between 7 and 9 years, while malignant tumours were more common in older dogs (Goldschmidt et al. 2017). In our study, the peak incidence of mammary tumours occurred at 8 years of age. The histologic patterns of mammary gland tumours in the dogs we examined were highly variable. In most cases, cancer developed from the ductal epithelium, accounting for 46.4% of all the malignant mammary gland tumours. Among the benign mammary gland tumours, we predominantly observed nodular fibrous cystic mastopathy, characterised by varying degrees of epithelial and connective tissue proliferation, and hyperplasia and dysplasia of cellular elements. Thus, our analyses underscore the importance of further research to analyse the impact of various risk factors associated with the occurrence of mammary tumours in dogs and humans.
